# Dexmedetomidine vs. lidocaine for postoperative analgesia in pediatric patients undergoing craniotomy: a protocol for a prospective, randomized, double-blinded, placebo-controlled trial

**DOI:** 10.1186/s13063-021-05774-9

**Published:** 2021-11-13

**Authors:** Yuan Zhang, Di Bao, Dongmei Chi, Lu Li, Bin Liu, Di Zhang, Lanxin Qiao, Yi Liang, Yaxin Wang, Xu Jin

**Affiliations:** Department of Anesthesiology, Beijing Tiantan Hospital, Capital Medical University, Beijing, 100070 People’s Republic of China

**Keywords:** Dexmedetomidine, Lidocaine, Pediatric, Craniotomy, Postoperative analgesia

## Abstract

**Background:**

Postoperative pain is a common problem that occurs in pediatric patients following neurosurgery which may lead to severe complications. Dexmedetomidine is a commonly used adjuvant medicine in craniotomy owing to its sedative, amnestic, analgesic, and neuroprotective properties. Besides, studies suggest that lidocaine has similar effects on sedation, analgesia, and neuroprotection. Both two adjuvants can reduce postoperative pain after neurosurgery in adults. However, it is still unknown whether dexmedetomidine or lidocaine can reduce postoperative pain in children undergoing craniotomy, and if yes, which is a better medicine choice. Therefore, we aimed to compare the effect of dexmedetomidine vs. lidocaine on postoperative pain in pediatric patients after craniotomy.

**Methods/design:**

We will perform a randomized (1:1:1), double-blind, placebo-controlled, single-center trial. Children aged 1–12 years scheduled for craniotomy will be eligible for inclusion. The 255 recruited participants will be stratified by age in two strata (1–6 years and 7–12 years), and then each stratum will be equally randomized to three groups: group D (infusion of dexmedetomidine [intervention group]), group L (infusion of lidocaine [intervention group]), and group C (infusion of normal saline [control group]). Patients will be followed up at 1 h, 2 h, 4 h, 24 h, and 48 h after surgery. The primary outcome will be total sufentanil consumption within 24 h after surgery.

**Discussion:**

In this clinical trial, we expect to clarify and compare the postoperative analgesic effect of dexmedetomidine vs. lidocaine infusion on pediatric patients undergoing craniotomy. We believe that the results of this trial will provide more choices for postoperative analgesia for the pediatric population.

**Trial registration:**

Chinese ClinicalTrials.gov ChiCTR1800019411. Registered on 10 November 2018

## Background

Acute postoperative pain is an unpleasant feeling and emotional experience caused by surgical trauma and is understood to influence pediatric patients’ emotions, behavior, and recovery after surgery, thereby further affecting morbidity and mortality. Therefore, proper perioperative analgesia management is of great significance to improve pediatric patients’ prognoses and attain a better therapeutic effect.

Unfortunately, postoperative pain in children has been seriously neglected for long, especially after neurosurgeries, and their pain response may be enhanced due to inadequate analgesia [1]. The likely reasons may include the following: (1) assessment of pain in children is often challenging and complicated, because children cannot verbalize their experience well, and (2) indications for administration of certain analgesics in children have not been well established, which further aggravates doctors’ concerns about these medicines’ side effects. To our best knowledge, only a limited number of studies have focused on pain after neurosurgery. Previous evidences have shown that approximately 60% of post-craniotomy patients experience moderate-to-severe pain for a short period postoperatively [2–5]. Besides, Teo et al. reported that 42% of children experienced at least one episode of moderate pain [6] within 72 h of the operation, and the postoperative pain may further result in children’s agitation, intracranial hypertension, epileptic seizures, and even postoperative hematoma, further affecting morbidity and mortality [7–10]. High-quality perioperative analgesia is significant to alleviate postoperative pain and can improve the quality of life and rehabilitation efficacy for pediatric patients undergoing neurosurgeries.

Commonly, perioperative analgesia includes opioid and non-opioid agents. Opioid administration is associated with serious risks and is a cause of concern with postoperative use in pediatric patients [11]. Various efforts have been taken to reduce opioid-related adverse effects and provide better pain control. Systemic dexmedetomidine or lidocaine as an adjuvant to general anesthesia may be a reliable option [12, 13].

Dexmedetomidine is a highly selective α2-adrenergic receptor agonist commonly used in neurosurgery [14]. The advantages of dexmedetomidine include reducing perioperative catecholamine to maintain intraoperative hemodynamic stability and exerting the unique neuroprotective effects by inhibiting the release of glutamate, pro-apoptotic proteins, and pro-inflammatory cytokines [14–17]. In addition, previous studies have shown that it can reduce sedative and opioid consumption and provide better analgesic effects with lesser concern for side effects than opioids [12, 18, 19]. For the above consideration, low-dose dexmedetomidine might be an interesting option for multimodal perioperative pain therapy in patients undergoing craniotomy. A meta-analysis performed by Schnabel et al. in 2013 suggested that compared with placebo, dexmedetomidine administration leads to reduced opioid consumption and decreased postoperative pain in adult patients undergoing various selective surgeries [20]. Similar conclusions were obtained for pediatric patients in the other meta-analysis that included 14 RCTs [21]. Besides, two more meta-analyses that focused on the effects of intraoperative dexmedetomidine on patients undergoing neurosurgeries also found dexmedetomidine, compared with placebo, could decrease perioperative opioid consumption and reduce postoperative pain intensity [19, 22]. Although the application of dexmedetomidine in pediatric is increasing, there are limited studies yet on the analgesic effect of dexmedetomidine in pediatric patients undergoing craniotomy [23–25].

Previous studies had shown that systemic infusion of lidocaine has effects on sedation, analgesia, neuroprotection, and maintaining hemodynamic stability [26–28]. Lidocaine can antagonize the *N*-methyl-d-aspartate receptor to provide analgesic effects and reduce opioid consumption with lower concern for side effects [13, 29]. Continuous infusion of low-dose lidocaine has been increasingly used to reduce perioperative pain in children. Several studies have also suggested that intravenous administration of lidocaine during operation could reduce postoperative pain intensity and decrease opioid consumption in children undergoing selective surgeries [30–33]. Besides, Peng et al. reported that compared with placebo, lidocaine could reduce acute postoperative pain in adults after supratentorial tumor surgery [22]. However, the analgesic effects of intravenous lidocaine in pediatric patients undergoing craniotomy require further investigation.

Dexmedetomidine and lidocaine are common adjuvant medicine anesthetics during operation for the sedative, analgesic, and neuroprotective properties. Besides, some studies have proved that both are effective in relieving postoperative pain in adults and children [34–38]. However, there are currently only few studies comparing the effects of both medicines on postoperative analgesia, and the conclusions are inconsistent [39, 40]. In particular, there are very limited studies comparing the effects of the two adjuvant analgesics on postoperative pain in children undergoing craniotomy. In addition, previous studies have shown that the two adjuvant analgesics can reduce the intraoperative consumption of sedatives and opioids [41, 42].

It is very important to identify the intensity of postoperative pain; however, some very young children cannot correctly express their pain intensity. At present, there is no ideal assessment scale for all pain types in children. The Face, Legs, Activity, Crying, Consolability (FLACC) scale, Wong–Baker Faces Pain-rating Scale (FACES), and numerical rating scale (NRS) are the common scales for children’s assessment. The former two scales are visualized assessment scale obtained by the observers, which are suitable for children aged 1–12 years, while NRS can only be applied for children aged 7–12 years owing to the need to children actively report pain levels. Considering the sedative effect of dexmedetomidine, the postoperative sedation status of participants should be evaluated. The Ramsay Sedation Score has been proven to satisfactorily reflect the children’s postoperative sedation conditions [43].

With this background, this randomized, double-blind, placebo-controlled, single-center trial has been designed to explore and compare the postoperative analgesic effects of intravenously administered dexmedetomidine, lidocaine, and normal saline on pediatric patients who are scheduled to undergo selective craniotomy.

## Methods

### Trial design and aim

This study is a single institution, stratified randomized, double-blind clinical trial with three parallel arms. The 255 recruited participants will be stratified by age into two strata (1–6 years and 7–12 years), and each stratum will be randomized on the basis of 1:1:1 into three groups.

**Fig. 1:**
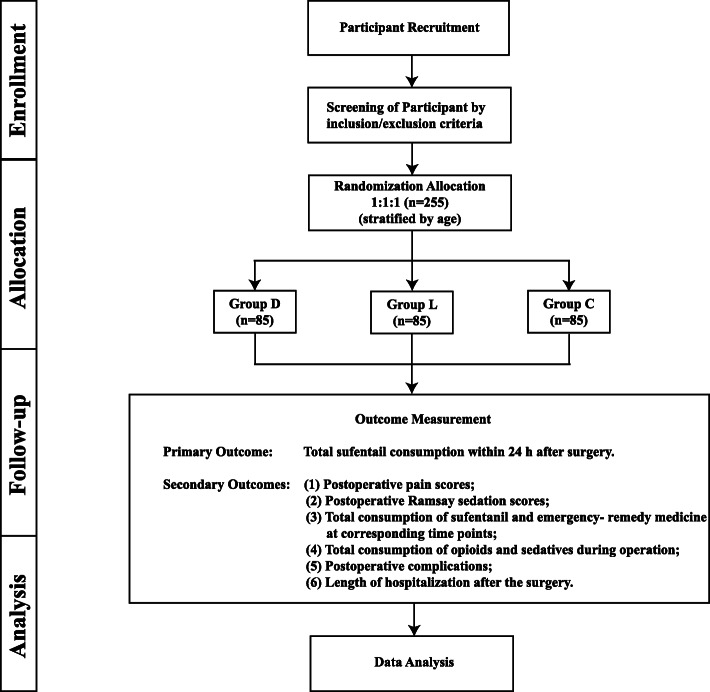
CONSORT flow diagram of the trial

We aim to clarify and compare the postoperative analgesic effect of dexmedetomidine vs. lidocaine on pediatric patients undergoing craniotomy. This study has been planned and designed according to the updated Consolidated Standards of Reporting Trials (CONSORT) statement [44]. The CONSORT diagram of this trial is shown in Fig. 1, and the trial schedule is shown in Table 1.

**Table 1:**
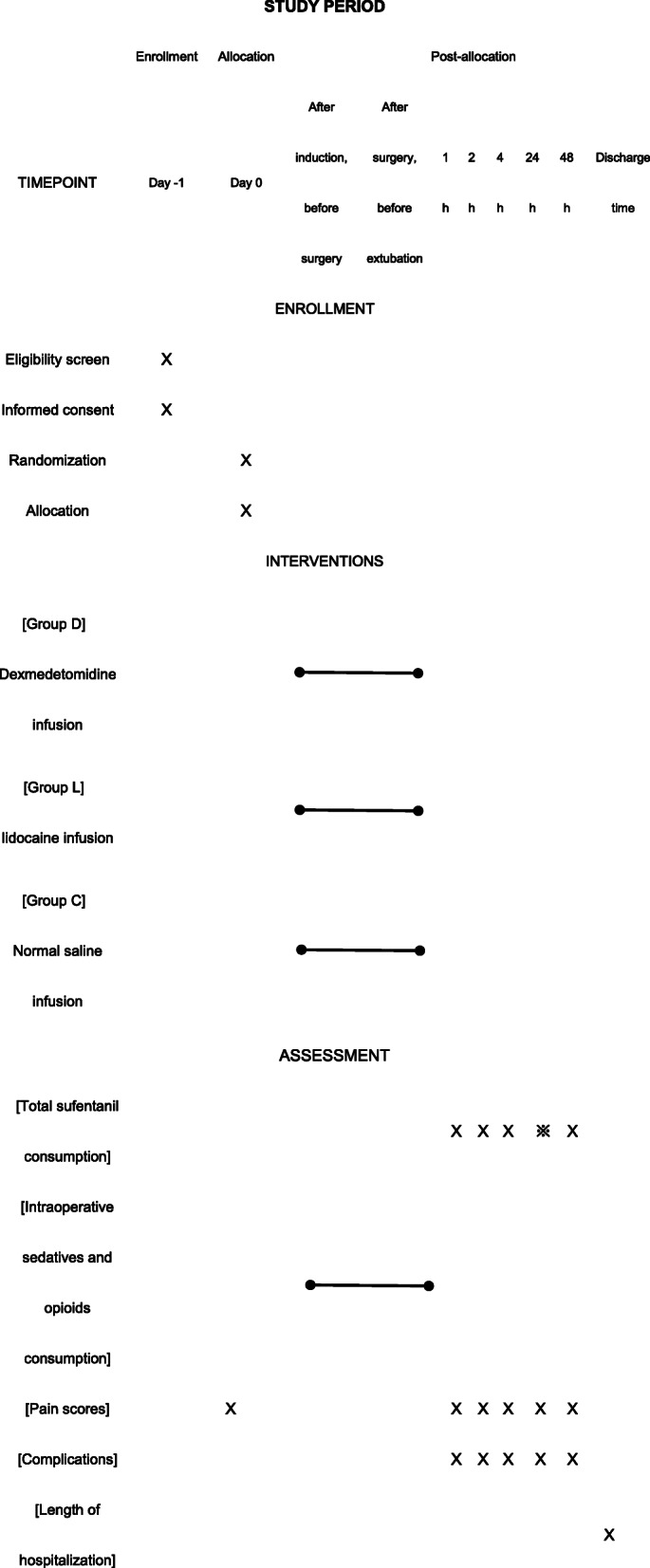
The schedule of enrollment, interventions, and assessments

### Study settings

All patients in this study will be consecutively screened and recruited in Beijing Tiantan Hospital, Capital Medical University, and the target completion date is November 2021.

### Composition, roles, and responsibilities for overseeing the study

The coordination center (CC) will comprise the principal investigator (PI), two anesthesiologists, two neurosurgeons, and an independent statistician. The responsibilities of the CC are coordinating and overseeing each stage of the trial including the recruitment, preoperative evaluation, perioperative intervention, postoperative follow-up visit, and analysis process to ensure study completion. The Trial Steering Committee (TSC) will include the PI, three anesthesiologists for pain management, two neurosurgeons, two neurosurgery nurses, and a statistician. The TSC will be responsible for training all the researchers involved in the trial and overseeing the review and approval of publications and presentations. An independent data monitoring committee (DMC) including two statisticians and a clinician will be formed to independently review the data security and accuracy of participants.

### Eligibility criteria

Young children (1–12 years of age) undergoing elective craniotomy in Beijing Tiantan Hospital, Capital Medical University, will be screened for eligibility. The inclusion criteria and exclusion criteria are listed below.

#### Inclusion criteria

The following are the inclusion criteria:

1. Aged between 1 and 12 years old.
2. Patients scheduled for elective craniotomy.
3. American Society of Anesthesiologists (ASA) physical status of I–III.
4. Participants and their authorized surrogates can provide informed consents.

#### Exclusion criteria

The following are the exclusion criteria:

1. History of psychological disease
2. Airway abnormalities, reactive airway diseases, or other respiratory diseases
3. Children with a known or suspected allergy and sensitivity or contraindications to intervention medicines (dexmedetomidine or lidocaine)
4. Liver or kidney dysfunction (when alanine aminotransferase, aspartate aminotransferase, blood urea nitrogen, and creatinine levels are ≥ 1.5 times the reference value)
5. Combined with atrioventricular block disease
6. Patients who have been included in other clinical trials
7. Children who cannot be extubated after surgery

### Patient screening and baseline management

Trained researchers will screen patients according to the eligibility criteria to participate in this study. Before enrollment, researchers need to explain the details to the participants and their authorized surrogates, including the benefits, risks, objectives, procedures, and other related issues, and ask them to sign an informed consent. Informed consent for patients aged 1–6 years will only be obtained from their authorized surrogates, while those for patients aged 7–12 years will be obtained both by the patient themselves and their authorized surrogates. Both dexmedetomidine and lidocaine are commonly used clinical medicines, and hence no potential harm, compensation, financial, or non-financial incentives are anticipated for the enrolled participants. Eligible patients will not be included until written informed consent is obtained. Baseline information about patients’ age, height, weight, ASA physical status, preoperative pain rating scores, laboratory findings, and surgical variables will be collected by study researchers.

### Randomization, allocation, masking, and blinding

Eligible participants will be stratified by age into two strata (age 1–6 years and 7–12 years) and then randomized in a 1:1:1 ratio for each group. The randomized allocation information after stratification will be determined by the computer-generated random number sequence through the Stata 15.1 software (Stata Corp, College Station, TX, USA). This operation would be completed by a researcher who will not participate in the study. The allocation information will be placed in an opaque-sealed envelope with a serial number. A research assistant who will not participate in anesthesia management, data collection, or any follow-up visit will open this envelope and prepare the study medicines and label them as “study medicine” for double-blinding. The study medicines including dexmedetomidine, lidocaine, and normal saline will be made into 50-mL volumes. The concentration of dexmedetomidine and lidocaine would be 4 μg mL^−1^ and 8 mg mL^−1^, respectively. The group assignment will remain blinded to related anesthesiologists, patients, and outcome assessors until they complete the follow-up visit for the last enrolled participant. Furthermore, adverse events (AEs) or severe adverse events (SAEs) leading to prolonged hospital stay or death will be recorded by responsible doctors or nurses and reported to the PI, and the study groups will be revealed following consultation with the PI.

### Intervention

The 255 eligible patients will be stratified by age into two strata (age 1–6 years and age 7–12 years), and then each stratum will be randomly allocated to three groups at a 1:1:1 ratio: dexmedetomidine group (group D, *n* = 85), lidocaine group (group L, *n* = 85), and normal saline group (group C, *n* = 85) according to the random number sequence. The intervention medicines will be administrated to patients immediately after tracheal intubation by the blinded anesthesiologist in charge. Infusion speed within the first 15 min will be calculated according to the following formula: 6 × body weight/4 mL h^−1^. The following infusion speed will be calculated as body weight/8 mL h^−1^. For each group, participants will receive intervention as follows:

Group D: Dexmedetomidine will be administrated at 1 μg kg^−1^ for 15 min after tracheal intubation, followed by 0.5 μg kg^−1^ h^−1^ infusion until the sutures of the endocranium are completed in group D.

Group L: Lidocaine will be administrated at 2 mg kg^−1^ for 15 min immediately after tracheal intubation, then followed by 1 mg kg^−1^ h^−1^ infusion until the sutures of endocranium are completed in group L.

Group C: Normal saline will be administrated intravenously immediately after tracheal intubation until the sutures of the endocranium are completed. The infusion speed within the first 15 min is calculated as 6 × body weight/4 mL h^−1^, and the following infusion speed will be calculated as body weight/8 mL h^−1^.

There will be no special criteria for modifying the interventions. If the AEs or SAEs appear during operation, they will be reported to PI and the inventions will be terminated.

### Anesthesia management

Routine monitoring of electrocardiography, non-invasive blood pressure measurement with 5-min interval, pulse oximetry saturation (SpO_2_), and end-tidal carbon dioxide partial pressure (P_ET_CO_2_) will be started when the patients arrive in the operating room. Midazolam (0.025–0.075 mg kg^−1^) will be used intravenously as premedication before anesthesia induction. If participating children are too nervous, irritable, or frightened to allow venous access, a dose of 0.5 mg kg^−1^ midazolam through the oral pathway will be administrated to relieve their anxiety.

Anesthesia will be induced with 0.3–0.5 μg kg^−1^ sufentanil, 1.5–2.5 mg kg^−1^ propofol, and 0.15 mg kg^−1^ cisatracurium. For children < 5 years of age with excessive anxiety, 6–8% sevoflurane will be administered through the inhalation pathway to induce anesthesia. After tracheal intubation, anesthesia will be maintained with a total intravenous infusion of 6–8 mg kg^−1^ h^−1^ propofol and 0.2–0.3 μg kg^−1^ min^−1^ remifentanil according to the responsible anesthesiologists’ decisions. Mechanical ventilation will be a volume-controlled mode with a tidal volume set at 8–10 ml kg^−1^, and the fraction of inspired oxygen (F_i_O_2_) set at ≥ 0.6, to a target SpO_2_ of 100%. The respiratory rate needs to be adjusted for the purpose of maintaining the P_ET_CO_2_ between 35 and 45 mmHg. About 30 min before the end of the surgery, 0.1 μg kg^−1^ sufentanil will be administered intravenously. Corresponding treatments will be applied to maintain the mean arterial pressure and heart rate within 30% of the baseline values such as supplying blood volume or using vasoactive agents. The temperature of each participant will be monitored and maintained at 35–37 °C during the whole operation. There will be no other additional analgesics administered during surgery; furthermore, scalp nerve blockade and local infiltration anesthesia to the incision will not be applied. The infusion of dexmedetomidine and lidocaine will be discontinued when the sutures of the endocranium are completed, and all other anesthetics will be discontinued at the end of surgery. The children will then be transferred to the postoperative anesthesia care unit (PACU), inpatient ward, or intensive care unit (ICU), as decided by the anesthesiologists unrelated to this study.

Further, an electronic analgesia pump (Apona® electronic infusion pump ZZB-I-150, APON Medical Technology Co., Ltd., Jiangsu, China) with sufentanil and ondansetron will be routinely used for postoperative pain and postoperative nausea and vomiting (PONV). The devices will be loaded with 2 μg kg^−1^ sufentanil and 0.3 mg kg^−1^ ondansetron diluted in 100 mL of normal saline. No background medicines will be administrated. The pump will only provide a bolus dose infusion when the button of the electronic analgesia pump is pressed (0.5 mL, 30-min lock-out time). Nurses in the PACU, ICU, and ward will be required for children aged 1–6 years to help them control the devices, and the button will be pressed when the FLACC score is > 4. Children aged 7–12 years will be administered analgesia with the help of the nurse until they are able to operate the button by themselves. When the FLACC pain score is > 5 and the FACES pain score is > 6, those children with insufficient analgesia will be administered acetaminophen at 15 mg kg^− 1^ orally, with enhanced single-bolus dose infusion of the electronic analgesia pump as a remedial procedure. The parameters of the electronic analgesia pump (including the total consumption of sufentanil and the number of compressions), and whether emergency rescue measures have been initiated (including the medicines’ dosage and frequency) will be recorded within 48 h after surgery.

### Outcome measurement

The primary outcome will be total sufentanil consumption within 24 h after surgery.

The secondary outcomes include the following aspects:

1. The postoperative pain intensity assessed by a series of pain rating scales (FLACC, FACES, and NRS) at 1 h, 2 h, 4 h, 24 h, and 48 h after surgery. Besides, the NRS scale is only applicable to children aged 7–12, but the FLACC and FACES scales are suitable for children aged 1–12 years. We will also classify and record the pain level of patients according to the scoring criteria of the three scales.
2. The postoperative sedation score (Ramsay) at 1, 2, 4, 24 and 48 h after surgery.
3. Total sufentanil consumption within 1, 2, 4, and 48 h after surgery and emergency remedy medicine consumption at 1, 2, 4, 24, and 48 h after surgery.
4. Total consumption of opioids and sedatives during operation.
5. Postoperative complications including bleeding, infection, neurological impairment, PONV, and severe adverse effects (SAEs) like disability and death, etc.
6. Length of hospitalization after surgery.

### Data collection and management

Before surgery, patients’ baseline conditions need to be collected carefully including (1) demographic variables such as sex, age, weight, and height; (2) ASA physical status; (3) baseline pain and sedation rating scores; (4) laboratory parameters; and (5) surgery variables such as incision site, lesion site, lesion character, and whether ventriculoperitoneal shunt was performed before craniotomy.

During the anesthetic period, the following data will be collected: (1) heart rate, peripheral oxygen saturation, blood pressure, and electrocardiographic patterns; (2) administration of anesthetics such as propofol, remifentanil, and vasoactive agents, including medicine type, dose, and other information; (3) the total duration of surgery and several important time points including surgery-start time, surgery-end time, patient awake time, and extubation time; (4) quantity of liquid intake and output; and (5) transfer site (PACU, surgery ward, or ICU).

Follow-up visits will be conducted at 1 h, 2 h, 4 h, 24 h, and 48 h after surgery. The total sufentanil consumption; the pain rating scores with FLACC [45], FACES [46], and NRS scale [47], and Ramsay sedation score; the emergency-remedy medicine consumption; and the parameters of the electronic analgesia pump at the abovementioned time points will be collected. Postoperative complications including bleeding, infection, neurological impairment, PONV, and SAEs such as disability and death will also be collected in detail.

The blinded and trained researchers will follow up with the patient at 1 h, 2 h, 4 h, 24 h, and 48 h after surgery and collect the outcome data. The blinded anesthesiologists in charge will record the patients’ intraoperative data. The researchers in charge of patient screening will collect the baseline data. Each participant will be assigned a unique identifier, and no personal information will be revealed. The research data will be double entered into the database by two independent data managers and checked regularly. The data will be hidden from all the researchers involved until the study is completed and the final data submitted for statistical analysis. Any biased and defective cases will be retained, except for those that cannot complete the primary outcome assessment, owing to death or discharge within 24 h. All the participants with deviations and deficiencies will be retained except for the cases that are unable to complete the assessment of the primary outcome owing to death or discharge within 24 h. The DMC will be responsible for monitoring the data, and the CC will review the trial conduct once for every 30 patients included. The TSC and IRB will review the research conduct throughout the trial period.

To protect confidentiality, the paper information will be stored in a locked cabinet inside the locked research office, and the electronic information will be kept in a password-protected electronic database. Special privilege to access the database to acquire personal information will require the consent of both the PI and DMC. All the information about the identification of participants used for data disclosure or statistical analysis will only appear as code numbers.

### Sample size calculation

It has been reported that the incidence of postoperative pain in adult patients undergoing elective craniotomy is about 60% [2–5]. The incidence of postoperative pain in children undergoing elective craniotomy will not be lower than that of adults; thus, we provisionally considered 60% as the positive rate in children’s postoperative pain for insurance purposes. We conservatively assume the study interventions could reduce the incidence of postoperative pain by half. The corrected alpha level was 0.0167 and the beta value was 0.1, and the three groups were compared in pairs. Based on an additional dropout rate of 10%, we calculated the total sample size required to be 255 patients (85 patients per arm) [36].

To recruit this number of participants, a 3-year inclusion period is anticipated according to the turnover rates of our hospital.

### Statistical analysis

The statistical analysis will be performed using the SPSS 23.0 software (International Business Machines Inc., USA). The continuous variables will be presented as mean ± standard deviation (*x̄* ± s) or interquartile range (IQR, 25–75% percentile), and nominal factors will be presented as number (proportion, %). All tests will be two-tailed and conducted at a 5% level of significance. Kolmogorov–Smirnov tests will be performed to detect the normal distribution of continuous variables. The *t*-test or analysis of variance (ANOVA) will be used for categorical variables with normal distribution and equal variance. The non-parametric test will be used appropriately for categorical variables with non-normal distribution and unequal variance. The chi-squared and Fisher’s exact tests will be used to compare proportions. Then, ANOVA or Kruskal–Wallis tests will be conducted for the intergroup comparisons of the primary outcome and other continuous variables among the three groups; if there are differences, further comparisons will be performed between any pair of study groups. Nominal variables will be compared using the chi-squared and Fisher exact tests. *p* < 0.05 will be considered to indicate statistical significance. The repeated measurements for general linear models and multivariate analysis will be conducted for the pain scores obtained from the three scales. All participants in the randomization will be analyzed, except for those whose primary outcome is missing, and multiple imputation will be conducted if this unintended missing data are more than 10%.

### Dissemination

The study protocol has been registered and is available on the Chinese Trial Registry website (registered in ChiCTR.org with the identifier ChiCTR1800019411). The results will be disseminated to all participants, researchers, and healthcare providers through study summary documents, courses, presentations, and the Internet. The datasets analyzed during the current study will be available from the corresponding author upon reasonable request.

## Discussion

Systemic administration of dexmedetomidine or lidocaine as an adjuvant to general anesthesia to relieve postoperative analgesia in adult patients undergoing elective surgeries is a reliable option as suggested by previous studies [12, 13, 18, 19, 29]. However, related data in pediatric patients is still lacking, especially with respect to children’s poor expression and safety profile of medicines [1]. This is a relatively large, stratified, randomized, double-blind, placebo-control, and single-center trial designed to determine the postoperative analgesic effects of systemic administration of dexmedetomidine or lidocaine in children undergoing craniotomy. The patients will be intravenously administered dexmedetomidine, lidocaine, or normal saline as control according to the randomized allocation. The efficacy and safety issues concerning the effect of dexmedetomidine or lidocaine on postoperative analgesia in pediatric patients will be tested. And the results will provide more choices for the multimodal perioperative pain therapy of children.

There are several reasons to explain why the setting proposed in the present protocol is appropriate. First, dexmedetomidine and lidocaine are both promising anesthetic adjuvants in pediatric patients undergoing craniotomy. The normal saline group is supposed to be the placebo group. Such settings could help us understand whether the intervention medicines (dexmedetomidine and lidocaine) are effective and which is the preferred adjuvant choice to reduce postoperative pain. Second, previous studies have proved that the administration of lidocaine and dexmedetomidine in this protocol is clinically safe and common for children undergoing craniotomy [30, 48–50]. Thus, this study will not increase the risk for children administered these two adjuvant analgesics. Third, this prospective, stratified, randomized, double-blinded, placebo-controlled trial with three parallel arms ensures the same conditions for the two intervention groups and the control group to facilitate comparisons between any pair of study groups to determine the effect of dexmedetomidine and lidocaine.

We acknowledge that this study protocol could have potential limitations. First, we only studied the clinically common dose of the intervention medicines rather than compare the effects of different concentrations. Second, this is a single-center study which may increase the inherent potential bias. However, our sample size is as large as possible to reduce the potential bias from this aspect. Nevertheless, the potential bias influenced by the skills of surgeons might be decreased given that Beijing Tiantan Hospital is ranked first for neurosurgery in China.

In the present protocol, we have tried to reduce bias as much as possible. We expect to conclusively determine the postoperative analgesic effects of intravenous infusion of dexmedetomidine and lidocaine in children undergoing craniotomy. This trial will provide more choices for the multi-model analgesia of pediatric patients undergoing neurosurgery and probably benefit prognosis.

## Trial status

The version number of this Clinical Research Program is V1.2. The trial began on December 1, 2018, and the recruitment will be completed approximately on November 30.

## Data Availability

The material of this study will be conserved in a secure repository at the Anesthesiology Department, Beijing Tiantan Hospital, Capital Medical University. Datasets will be available from the PI upon reasonable request.

## Abbreviations

FLACC: Face, Legs, Activity, Crying, Consolability
FACES: Wong–Baker Faces Pain-Rating Scale
NRS: Numerical rating scale
CONSORT: Consolidated Standards of Reporting Trials
CC: Coordination center
PI: Principal investigator
TSC: Trial Steering Committee
DMC: Data monitoring committee
ASA: American Society of Anesthesiologists
AEs: Adverse events
SAEs: Severe adverse events
SpO_2_: Pulse oximetry saturation
F_i_O_2_: Fraction of inspired oxygen
P_ET_CO_2_: End-tidal carbon dioxide partial pressure
PACU: Postoperative anesthesia care unit
ICU: Intensive care unit
PONV: Postoperative nausea and vomiting

## References

[1] Dunbar PJ, Visco E, Lam AM (1999). Craniotomy procedures are associated with less analgesic requirements than other surgical procedures. Anesthesia and Analgesia..

[2] Flexman AM, Ng JL, Gelb AW (2010). Acute and chronic pain following craniotomy. Curr Opin Anaesthesiol..

[3] Gottschalk A, Berkow LC, Stevens RD, Mirski M, Thompson RE, White ED (2007). Prospective evaluation of pain and analgesic use following major elective intracranial surgery. Journal of Neurosurgery..

[4] Mordhorst C, Latz B, Kerz T, Wisser G, Schmidt A, Schneider A (2010). Prospective assessment of postoperative pain after craniotomy. J Neurosurg Anesthesiol..

[5] Silberstein S, Olesen J, Bousser M, Diener H, Dodick D, First M (2005). International Headache Society. The International Classification of Headache Disorders, (ICHD-II)—revision of criteria for 8.2 medication-overuse headache. Headache..

[6] Teo JH, Palmer GM, Davidson AJ (2011). Post-craniotomy pain in a paediatric population. Anaesth Intensive Care..

[7] Breivik H, Stubhaug A (2008). Management of acute postoperative pain: still a long way to go!.

[8] Carr DB, Goudas LC (1999). Acute pain. Lancet..

[9] De Benedittis G, Lorenzetti A, Migliore M, Spagnoli D, Tiberio F, Villani RM (1996). Postoperative pain in neurosurgery: a pilot study in brain surgery. Neurosurgery..

[10] Guy J, Hindman BJ, Baker KZ, Borel CO, Maktabi M, Ostapkovich N (1997). Comparison of remifentanil and fentanyl in patients undergoing craniotomy for supratentorial space-occupying lesions. Anesthesiology..

[11] Duedahl TH, Hansen EH (2007). A qualitative systematic review of morphine treatment in children with postoperative pain. Paediatr Anaesth..

[12] Hall JE, Uhrich TD, Barney JA, Arain SR, Ebert TJ (2000). Sedative, amnestic, and analgesic properties of small-dose dexmedetomidine infusions. Anesth Analg..

[13] Koppert W, Weigand M, Neumann F, Sittl R, Schuettler J, Schmelz M (2004). Perioperative intravenous lidocaine has preventive effects on postoperative pain and morphine consumption after major abdominal surgery. Anesthesia and Analgesia..

[14] Cormack JR, Orme RM, Costello TG (2005). The role of α2-agonists in neurosurgery. Journal of Clinical Neuroence..

[15] Dahmani S, Rouelle D, Gressens P, Mantz J (2010). Characterization of the postconditioning effect of dexmedetomidine in mouse organotypic hippocampal slice cultures exposed to oxygen and glucose deprivation. Anesthesiology..

[16] Gao J, Sun Z, Xiao Z, Du Q, Niu X, Wang G (2019). Dexmedetomidine modulates neuroinflammation and improves outcome via alpha2-adrenergic receptor signaling after rat spinal cord injury. Br J Anaesth..

[17] Degos V, Charpentier TL, Chhor V, Brissaud O, Lebon S, Schwendimann L (2013). Neuroprotective effects of dexmedetomidine against glutamate agonist-induced neuronal cell death are related to increased astrocyte brain-derived neurotrophic factor expression. Anesthesiology..

[18] Bajwa S, Kulshrestha A (2013). Dexmedetomidine: an adjuvant making large inroads into clinical practice. Annals of medical and health sciences research..

[19] Liu Y, Liang F, Liu X, Shao X, Jiang N, Gan X (2018). Dexmedetomidine reduces perioperative opioid consumption and postoperative pain intensity in neurosurgery: a meta-analysis. J Neurosurg Anesthesiol..

[20] Schnabel A, Meyer-Friessem C, Reichl S, Zahn P, Pogatzki-Zahn E (2013). Is intraoperative dexmedetomidine a new option for postoperative pain treatment? A meta-analysis of randomized controlled trials. PAIN®.

[21] Le AB, Michelet D, Hilly J, Maesani M, Dilly M, Brasher C (2015). Efficacy of intraoperative dexmedetomidine compared with placebo for surgery in adults: a meta-analysis of published studies.

[22] Peng K, Wu SR, Liu HY, Ji FH (2014). Dexmedetomidine as an anesthetic adjuvant for intracranial procedures: meta-analysis of randomized controlled trials. Journal of Clinical Neuroscience..

[23] Phan H, Nahata MC (2008). Clinical uses of dexmedetomidine in pediatric patients. Paediatr Drugs..

[24] Sheshadri V, Chandramouli BA (2016). Pediatric awake craniotomy for seizure focus resection with dexmedetomidine sedation-a case report. J Clin Anesth..

[25] Song I, Yi S, Lim H, Lee J, Kim E, Cho J (2019). A population pharmacokinetic model of intravenous dexmedetomidine for mechanically ventilated children after neurosurgery. Journal of clinical medicine.

[26] Lei B, Cottrell JE, Kass IS (2001). Neuroprotective effect of low-dose lidocaine in a rat model of transient focal cerebral ischemia. Anesthesiology..

[27] Lei B, Popp S, Capuano-Waters C, Cottrell JE, Kass IS (2002). Effects of delayed administration of low-dose lidocaine on transient focal cerebral ischemia in rats. Anesthesiology..

[28] Chen K, Wei P, Zheng Q, Zhou J, Li J (2015). Neuroprotective effects of intravenous lidocaine on early postoperative cognitive dysfunction in elderly patients following spine surgery. Med Sci Monit..

[29] Sugimoto M, Uchida I, Mashimo T (2003). Local anaesthetics have different mechanisms and sites of action at the recombinant N-methyl-D-aspartate (NMDA) receptors. Br J Pharmacol..

[30] Ayulo MA, Phillips KE, Tripathi S (2018). Safety and efficacy of IV Lidocaine in the treatment of children and adolescents with status migraine. Pediatric critical care medicine..

[31] Blanda M, Rench T, Gerson LW, Weigand JV (2001). Intranasal lidocaine for the treatment of migraine headache: a randomized, controlled trial. Acad Emerg Med..

[32] Maizels M, Scott B, Cohen W, Chen W (1996). Intranasal lidocaine for treatment of migraine: a randomized, double-blind, controlled trial. JAMA..

[33] Mooney JJ, Pagel PS, Kundu A (2014). Safety, tolerability, and short-term efficacy of intravenous lidocaine infusions for the treatment of chronic pain in adolescents and young adults: a preliminary report. Pain Med..

[34] Bellon M, Le Bot A, Michelet D, Hilly J, Maesani M, Brasher C (2016). Efficacy of intraoperative dexmedetomidine compared with placebo for postoperative pain management: a meta-analysis of published studies. Pain Ther..

[35] de Oliveira CMB, Coelho LMG, Valadao JA, Moura ECR, da Silva AAM, de Lima RC (2020). Assessment of the effect of perioperative venous lidocaine on the intensity of pain and IL-6 concentration after laparoscopic gastroplasty. Obes Surg..

[36] Grape S, Kirkham KR, Frauenknecht J, Albrecht E (2019). Intra-operative analgesia with remifentanil vs. dexmedetomidine: a systematic review and meta-analysis with trial sequential analysis. Anaesthesia..

[37] McCarthy GC, Megalla SA, Habib AS (2010). Impact of intravenous lidocaine infusion on postoperative analgesia and recovery from surgery: a systematic review of randomized controlled trials. Drugs..

[38] Weibel S, Jokinen J, Pace NL, Schnabel A, Hollmann MW, Hahnenkamp K (2016). Efficacy and safety of intravenous lidocaine for postoperative analgesia and recovery after surgery: a systematic review with trial sequential analysis. Br J Anaesth..

[39] Sherif AA, Elsersy HE (2017). The impact of dexmedetomidine or xylocaine continuous infusion on opioid consumption and recovery after laparoscopic sleeve gastrectomy. Minerva Anestesiol..

[40] Xu SQ, Li YH, Wang SB, Hu SH, Ju X, Xiao JB (2017). Effects of intravenous lidocaine, dexmedetomidine and their combination on postoperative pain and bowel function recovery after abdominal hysterectomy. Minerva Anestesiol..

[41] Hans GA, Lauwick SM, Kaba A, Bonhomme V, Struys MM, Hans PC (2010). Intravenous lidocaine infusion reduces bispectral index-guided requirements of propofol only during surgical stimulation. Br J Anaesth..

[42] Le Guen M, Liu N, Tounou F, Auge M, Tuil O, Chazot T (2014). Dexmedetomidine reduces propofol and remifentanil requirements during bispectral index-guided closed-loop anesthesia: a double-blind, placebo-controlled trial. Anesth Analg..

[43] Li A, Yuen VM, Goulay-Dufay S, Sheng Y, Standing JF, Kwok PCL (2018). Pharmacokinetic and pharmacodynamic study of intranasal and intravenous dexmedetomidine. Br J Anaesth..

[44] Schulz KF, Altman DG, Moher D (2010). CONSORT 2010 statement: updated guidelines for reporting parallel group randomised trials. BMC medicine..

[45] Merkel SI, Voepel-Lewis T, Shayevitz JR, Malviya S (1997). The FLACC: a behavioral scale for scoring postoperative pain in young children. Pediatr Nurs..

[46] Bieri D, Reeve RA, Champion GD, Addicoat L, Ziegler JB (1990). The Faces Pain Scale for the self-assessment of the severity of pain experienced by children: development, initial validation, and preliminary investigation for ratio scale properties. Pain..

[47] Instruments PI, Health NIo. Warren Grant Magnuson Clinical Center, July 2003. Archived from the original (PDF) on. 2012:09-14.

[48] Abram SE, Yaksh TL (1994). Systemic lidocaine blocks nerve injury-induced hyperalgesia and nociceptor-driven spinal sensitization in the rat. Anesthesiology.

[49] Jooste EH, Hammer GB, Reyes CR, Katkade V, Szmuk P (2017). Phase IV, Open-label, safety study evaluating the use of dexmedetomidine in pediatric patients undergoing procedure-type sedation. Front Pharmacol..

[50] Zuppa AF, Nicolson SC, Wilder NS, Ibla JC, Gottlieb EA, Burns KM (2019). Results of a phase 1 multicentre investigation of dexmedetomidine bolus and infusion in corrective infant cardiac surgery. Br J Anaesth..

